# Family and health professional perspectives of the CHD LIFE cardiac developmental long-term care pathway: a qualitative evaluation

**DOI:** 10.1038/s41390-025-04380-8

**Published:** 2025-09-30

**Authors:** Bridget Abell, Thomasina Donovan, Karen J. Eagleson, Robert Justo, Ben Auld, Sonia Riley, Steven M. McPhail

**Affiliations:** 1 Australian Centre for Health Services Innovation and Centre for Healthcare Transformation, School of Public Health and Social Work, Faculty of Health, Queensland University of Technology, Brisbane, QLD Australia; 2 Queensland Paediatric Cardiac Service, Queensland Children’s Hospital, Brisbane, QLD Australia; 3 Department of Occupational Therapy, Queensland Children’s Hospital, Brisbane, QLD Australia; 4 Digital Health and Informatics, Metro South Health, Brisbane, QLD Australia

## Abstract

**Background:**

This study evaluates the implementation and impact of the Congenital Heart Disease Long-term Improvements in Functional hEalth (CHD LIFE) decentralized care pathway in Australia. It explores perspectives of families and healthcare professionals, identifying key enablers, barriers, and opportunities for improvement.

**Methods:**

A qualitative evaluation was conducted using semi-structured interviews and reflexive thematic analysis. Purposeful and convenience sampling recruited (a) parents (*n* = 37) of children with CHD referred for developmental follow-up via the CHD LIFE pathway and (b) key service providers and organizational stakeholders (*n* = 23).

**Results:**

Findings were organized into implementation, perceived impact, and improvement summary domains. Families and providers generally found decentralization of the pathway acceptable and contextually appropriate, but not without challenges. *Implementation* was enabled by the pathway’s co-design, centralized foundations, and system alignment, but challenged by variability in local capacity, provider engagement, and substantial reliance on families. Perceived *impacts* were noted for providers, parents, and children, including capacity building. Stakeholders identified a range of tangible *improvements* to enhance equity and sustainability.

**Conclusions:**

CHD LIFE presents a promising decentralized, family-centered follow-up model improving access and supporting local care. Strengthening support mechanisms for families and providers remains essential to promote equity, reduce burden, and ensure sustainable implementation across diverse settings.

**Impact:**

- The CHD LIFE care pathway demonstrates the feasibility and acceptability of a decentralized neurodevelopmental follow-up model for children with congenital heart disease, reducing geographic and logistical barriers while promoting family-centered care.
- Findings contribute to the discourse around alternative care models, highlighting the barriers, enablers, and impacts of decentralized care implementation.
- The study also highlights the need for structured support systems, ongoing provider engagement, and more standardized processes to ensure equitable and sustainable care delivery.
- This work informs future policy and practice, offering a scalable model for cardiac neurodevelopmental follow-up, which may potentially be adaptable to other healthcare contexts.

## Introduction

Children with Congenital Heart Disease (CHD) are at risk of neurodevelopmental challenges, including developmental delays, and persistent learning, psychosocial, and behavioral difficulties.^1^ These ongoing concerns associated with CHD can also have broader impacts on families, peers, schools, and health and social services.^2–4^ Optimal care for children with CHD and their families should include integrated neurodevelopmental follow-up at key developmental stages.^5,6^ While this care is commonly provided via specialist-center-based programs, challenges have been noted with these programs in terms of accessibility, resourcing, staffing, out-of-pocket costs, and family travel burden.^7^ Additionally, these types of models may be sub-optimal for supporting long-term neurodevelopmental follow-up for children with CHD in regions with different geographical and healthcare contexts to the United States, where they were first established.^8^

Alternative models of neurodevelopmental follow-up include services provided within primary healthcare, through educational institutions, or in community settings to meet the needs of families supporting children with CHD.^6^ Such decentralized models of care delivery have potential for improving overall access to assessments and treatments and encouraging greater uptake of follow-up care. The Congenital Heart Disease Long-term Improvements in Functional hEalth (CHD LIFE) Program’s long-term developmental care pathway is one such decentralized care model. It was introduced in Queensland, Australia, to provide a state-wide integrated long-term care approach to developmental surveillance for children with CHD, which is embedded in primary care and local community services.^9,10^

Generating and sharing evidence about the implementation (including feasibility, acceptability, and accessibility) as well as the effectiveness of different models of neurodevelopmental follow-up care is a key priority in the field.^11^ Evaluations of models of care, however, have been scarce, including those that seek to understand the experiences of both family members and health professionals. Such studies have the potential to identify barriers and aid in improvements to the design and delivery of follow-up care. Consequently, the aim of this study was to generate evidence about the implementation and perceived impact of the CHD LIFE care pathway in Queensland, Australia, by examining the experiences and perspectives of families supporting children with CHD and health professional stakeholders involved in their neurodevelopmental follow-up care.

## Methods

### Study design

We conducted a qualitative evaluation study using semi-structured interviews of families and health professionals with reflexive thematic analysis to investigate the implementation, impacts, and experiences associated with the CHD LIFE care pathway in Queensland. Qualitative evaluation approaches provide insight into how complex interventions work in practice, allowing in-depth exploration of contextual factors and how they may be associated with outcomes.^12^ By focusing on both perceived outcomes and implementation processes, we aimed to understand both how well the pathway was working and why (barriers and enablers), allowing for the development of context-specific strategies for optimizing future pathway delivery. This study was part of a larger multi-year mixed-methods program of research examining, evaluating, and costing cardiac neurodevelopmental follow-up processes in Australia.

The Consolidated Criteria for Reporting Qualitative Research Checklist (COREQ) was used for transparent and complete reporting of methods (Additional File 1: Supplementary Table S1).^13^ Ethical approval was obtained from the Children’s Health Queensland Hospital and Health Service Human Research Ethics Committee (LNR/21/QCHQ/73748) prior to study commencement.

### Context

The study took place in Queensland, Australia. Children’s Health Queensland is a public health service supporting pediatric specialist care across the geographically large state. It includes the Queensland Children’s Hospital, a large metropolitan-based tertiary level facility providing cardiac surgery for all children in the State of Queensland.

#### Congenital Heart Disease Long-term Improvements in Functional hEalth (CHD LIFE) Program and long-term developmental care pathway

The Queensland Paediatric Cardiac Service (QPCS) CHD LIFE Program is a partnership program designed to “improve long-term functional health outcomes in children with CHD and their families by implementing best-practice recommendations, facilitating early intervention, driving advocacy and awareness, service improvement and capacity building, conducting research and training professionals.”^9^ A multidisciplinary leadership group, inclusive of consumer advocacy representation, oversees the program and drives initiatives, one of which is a developmental care pathway. The CHD LIFE care pathway is a decentralized model of care to support the long-term developmental follow-up of children across Queensland who undergo open-heart surgery at less than 12 months of age.^10^ The pathway was developed through a service translation initiative in partnership between the QPCS, healthcare improvement partners, consumers, and state-wide hospital and health services. It evolved to support access to services closer to home from a previously centralized model at the Queensland Children’s Hospital. The establishment of the centralized developmental follow-up program and transition to the CHD LIFE care pathway in 2018 has previously been reported.^9^

**Fig. 1:**
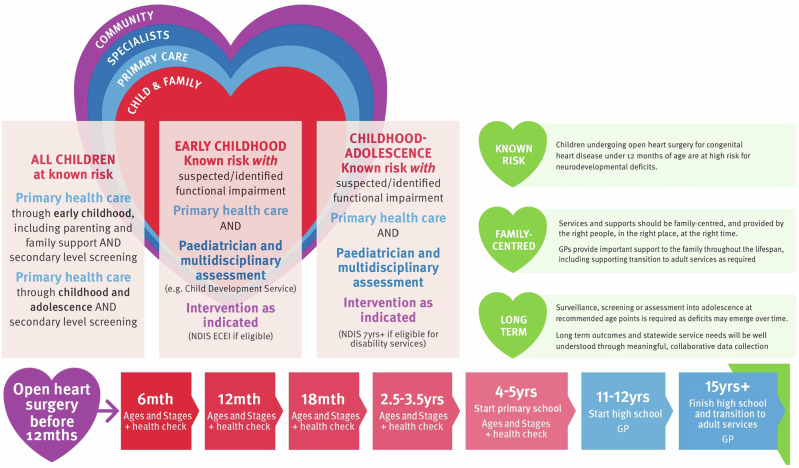
CHD LIFE cardiac developmental long-term care pathway (2018–present). Adapted from Eagleson et al.^9^ ASQ Ages and Stages Questionnaire, CHD LIFE Congenital Heart Disease Long-term Improvement in Functional hEalth, ECEI Early Childhood Early Intervention, GP general practitioner, NDIS National Disability Insurance Scheme. Pathway and companion document available from https://www.childrens.health.qld.gov.au/resources/health-professionals/committees-and-groups/qcycn/pink-book-developmental-needs-children-congenital-heart-disease.

The CHD LIFE care pathway aims to empower children at high risk for adverse neurodevelopmental outcomes and their families to access the right surveillance, evaluation, and treatments in the right location for their circumstances, at the right time, for optimal prevention and treatment for neurodevelopmental complications. It provides a multidisciplinary and integrated care model according to contemporary clinical recommendations.^14^ The pathway’s focus is on the child and family as the center of care and includes, as a minimum level of care, long-term secondary level screening in local primary care services at key time points with the Ages and Stages Questionnaire (ASQ-3)^15^ to support early identification and timely access to assessment and/or intervention. Care pathway recommendations are illustrated in Fig. 1. A range of strategies have been adopted to support early and ongoing pathway implementation, focused on using existing services in the local environment. These include an online resource, targeted health service engagement and education, promoting advocacy and awareness, capacity building, a centrally maintained database of eligible and consented children, reminder postcards and stickers, service referrals, electronic health record alerts, and ongoing parent education.

### Study participants and recruitment

The recruitment strategy was specific to each stakeholder group and had aspects of both purposive and convenience sampling. For both groups, the sample size was guided by a combination of maximum data variation across key participant demographics, richness of data to answer the research question, and recurrence of codes or categories in the interview data (thematic saturation).^16^ All interested participants independently contacted the university-based researchers (who differed from the clinical research team) with whom they had no pre-existing personal or professional relationships, ensuring that participation was voluntary and free from external pressure or expectations. The study coordinator then provided information, answered questions, arranged interviews, and obtained written consent. All participants were informed that participation was voluntary.

#### Family stakeholders

Eligible participants were parents or carers of children with CHD in Queensland who were referred for developmental follow-up via the CHD LIFE care pathway. This included those who were enrolled in the earlier centralized model of care and later transitioned to follow-up via the current decentralized model.

Potential participants were identified from the CHD LIFE program database, which represented approximately 80% of eligible families accessing the surgical center. All families on the database who had previously consented to being contacted by email for research purposes (84% families) were invited to participate in the research study by the clinical members of the research team. The invitation included a link to a video explaining the study (https://www.aushsi.org.au/research/projects/chd-life-family-information/), which featured both a clinician familiar to the families and a university-based researcher external to the CHD LIFE program. Participants received a $20 gift voucher as appreciation for their time (in line with the contemporary Consumer Health Queensland renumeration rates of $40 per hour for consumer participation in interviews lasting 30 min).

We aimed to capture a broad and representative sample of families, including those in urban and regional areas and of varying durations of engagement with the pathway/follow-up care. Consequently, after initial recruitment had been exhausted with replies, an additional round of targeted invitations was made using purposive sampling to ensure representation across key characteristics, such as geographic location.

#### Health professional stakeholders

Eligible participants were key service providers and organizational-level stakeholders involved in the planning, administration, leadership, implementation, or service delivery of the CHD LIFE care pathway and/or those involved more broadly in the care of children with CHD and their long-term neurodevelopmental needs in Queensland. A purposeful strategy was used to recruit participants across various geographical locations, clinical settings, clinical disciplines, and leadership levels.

Identification and recruitment of health professionals occurred via a range of strategies. The university-based project coordinator sent generic emails to child health and child development services across the state, as well as targeted invitations to key stakeholders within and external to Children’s Health Queensland who had been identified by the clinical researchers on the study team. The study was also advertised via email-based mailing lists of child development, child health, general practice, and pediatrics state-wide networks.

### Data collection

Virtual interviews were conducted from December 2022 to June 2023. Interviews were scheduled based on each participant’s preferred timing and modality (Microsoft Teams, Zoom, or phone). BA developed a semi-structured interview guide for each stakeholder group to capture their experiences, which was checked for face and content validity by the clinical research team. Two experienced PhD-qualified health service researchers (BA, female and DR, male) conducted the interviews. Interviews lasted between 20 and 60 min. Audio recordings of the interviews were professionally transcribed.

#### Family interviews

The interview guide for family stakeholders (Additional File 2) comprised several open-ended questions, divided into three sections: (1) their experiences with the CHD LIFE care pathway and developmental follow-up; (2) impact of the care pathway on their child and family; and (3) suggestions for care pathway improvements. Demographic, clinical, and care pathway enrollment details for each parent-child dyad were extracted from the CHD LIFE database.

#### Health professional interviews

The interview guide for health professional stakeholders (Additional File 3) comprised several open-ended questions, divided into four sections: (1) knowledge and experience with neurodevelopmental follow-up for CHD; (2) care pathway implementation, barriers and facilitators using the five domains of the Consolidation Framework for Implementation Research as prompts to ensure comprehensive exploration of implementation factors across a range of potential system levels^17^; (3) outcomes and impact of the care pathway on families and providers; (4) suggestions for pathway improvements and sustainability. Participants’ professions, locations, and other demographic characteristics were also collected.

### Data analysis

The transcripts were analyzed by two qualitative researchers (BA and TD) using reflexive thematic analysis.^18^ This analysis approach supports understanding people’s experiences, opinions, and behaviors, which is aligned with the aim of evaluating implementation of the CHD LIFE care pathway from multiple perspectives. Family and health professional transcripts were first analyzed as separate datasets in NVivo (Lumivero, release 14.23.2), with initial coding and theming undertaken within each dataset. These were then compared to identify commonalities and differences, and synthesized into common codes and themes for cross-perspective evaluation. To ensure rigor of the analysis process, BA and TD conducted reflective meetings to discuss analysis choices and emerging insights, and findings were contextualized and supported with quotes.

Analysis was iterative and inductive, involving a process of first closely reading transcripts to identify meaningful segments of data, which were then coded. A coding scheme was developed and continuously refined through discussion and reflection, structured around the two key aims of the study: (1) understanding participants’ experiences and perspectives of implementation, including perceived barriers and enablers; and (2) assessing the pathway’s perceived impact on families and health professionals. The coding tree was primarily organized by aim (i.e., implementation and impacts), with codes mapped diagrammatically and grouped into topically coherent themes within each aim/topic domain. BA collaborated with the clinical research team to review, reflect on, contextualize, and finalize the themes, ensuring they accurately reflected the complete dataset for both families and providers. Exemplar quotes were selected to illustrate themes and to preserve the richness and diversity of participant perspectives. In addition to identifying themes related to the primary aims, we remained open to unanticipated findings. As part of this, themes related to stakeholder-generated suggestions for improvement were noted, requiring a third topic domain to be added to the coding tree.

## Results

### Participant characteristics

**Fig. 2:**
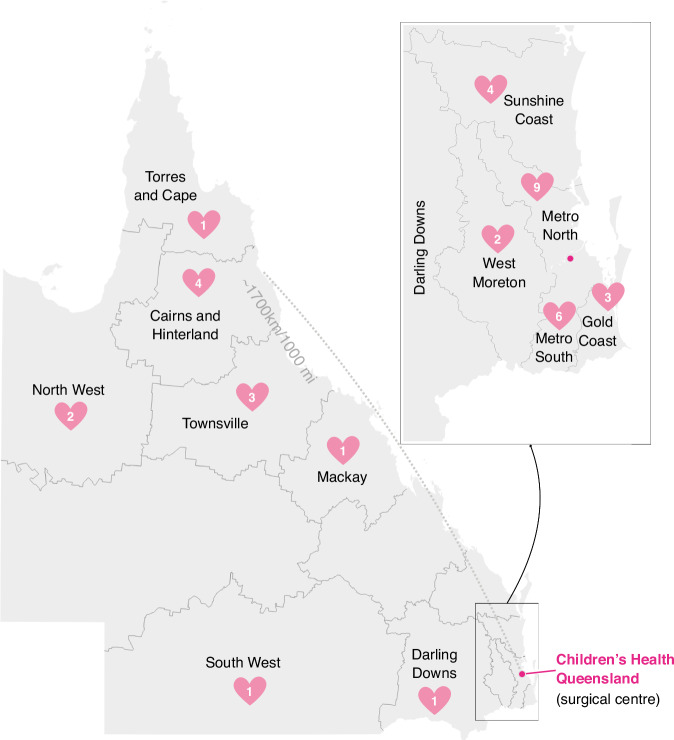
Geographical locations of family study participants. Number of parents (*n* = 37) in each Queensland hospital and health district at the time of the interview.

Sixty stakeholders participated in the study (62% parents, 38% health professionals). Of the 289 families invited, 37 parents completed phone interviews, representing 36 individual children (a mother and father of the same child were both interviewed). Almost all parents interviewed were mothers (*n* = 35, 95%) and were located across 10 of Queensland’s sixteen hospital and health services, spanning metropolitan, regional, rural, and very remote areas (Fig. 2). The parent cohort interviewed displayed high levels of educational attainment (73% of mothers and 65% of fathers had completed higher education). The characteristics of the 36 children (*n* = 18, 50% male) whose parents took part in the study are provided in Table 1. Six children had a genetic comorbidity. Three children initially accessed care in the original centralized program of service delivery before joining the decentralized pathway; the remainder accessed care via the current CHD LIFE care pathway.

**Table 1:** Characteristics of the participating parents (*n* = 37) and their child (*n* = 36)

Participant characteristics	Count
**Child’s gender**	
Female	18
Male	18
**Child’s age at time of interview**	
1–2 years old	8
2–3 years old	9
3–4 years old	6
4–5 years old	10
>5 years old	3
**Child is Aboriginal and/or Torres Strait Islander**	
Yes	4
No	32
**Residential location at time of discharge**^a^	
Metropolitan (MMM1)	22
Regional (MMM 2)	10
Remote community (MMM6)	2
Very remote community (MMM7)	2
**Diagnosis**	
Septal defect (SD)	10
Single ventricle physiology	4
Transposition of the great arteries	11
Tetralogy of Fallot	6
Valve stenosis	2
Other	3
**Genetic comorbidities**	
Yes	6
No/not reported	30
**Antenatal diagnosis**	
Yes	26
No	10
**Maternal education**	
High School not completed	2
High School	5
TAFE/Technical College	7
Tertiary	20
Not reported	2
**Paternal education**	
High School not completed	3
High School	8
TAFE/Technical College	8
Tertiary	16
Not reported	1
**HHS on discharge**	
Cairns & Hinterland	4
Metro North	7
Metro South	9
Darling Downs	2
Gold Coast	3
Mackay	2
North West	1
Sunshine Coast	2
Townsville	4
West Moreton	2
**Interviewee (Parent) gender**^b^	
Female	35
Male	2
**Initial CHD LIFE care pathway type**	
Centralized	3
Decentralized	34

^a^Modified Monash Model (MMM) classification according to geographical remoteness and town size.

^b^Two parents are interviewed for one child.

Twenty-three of the 90 invited health professionals participated in online interviews (Table 2). Participants represented a range of occupational roles, but most were women working in hospital-based, metropolitan health services. The majority reported a high level of experience with CHD and neurodevelopment, and at least some level of familiarity with the care pathway.

**Table 2:** Characteristics of the participating health professionals (*n* = 23)

Participant characteristics	Count
**Gender**	
Female	17
Male	6
**Position at organization**	
Nurse	3
Cardiologist	3
Pediatrician	3
Occupational Therapist	3
General Practitioner	2
Physiotherapist	2
Speech Pathologist	1
Other (manager, disability, research, advocacy, admin)	6
**Years of experience in the role**	
5–9 years	2
10–14 years	5
15+ years	6
Not stated	10
**Service location**	
Hospital-based	16
Community settings	5
Other (e.g., state-wide, education, research)	2
**Occupation location**^a^	
Metropolitan (MM 1)	19
Regional (MM 2)	3
Not applicable (state-wide)	1
**Experience with congenital heart disease**	
None	0
Some	2
High	14
Not stated	7
**Experience with neurodevelopment delays**	
None	0
Some	2
High	14
Not stated	7
**Familiarity with the CHD LIFE care pathway**	
None	1
Some	4
High	11
Not stated	7

^a^Modified Monash Model (MMM) classification according to geographical remoteness and town size.

### Overview of findings

Qualitative findings are organized into themes within three overarching summary domains. Two of these directly address the research questions about (1) implementation experiences and perspectives, and (2) perceived impacts of the CHD LIFE care pathway. Although the primary aim of this study was to explore the implementation and perceived impacts of the care pathway, analysis also identified a range of stakeholder-informed opportunities for improvement. These insights, while not an explicit focus of the study design, emerged consistently across interviews as suggestions to enhance the effectiveness, accessibility, and sustainability of the pathway. Consequently, they have been presented as themes in a third summary domain.

### Participants’ experiences and perspectives on the CHD LIFE pathway implementation

Eight themes were identified, which were common to both families’ and professionals’ experience of the care pathway, and an additional theme, which was noted by professionals only. These themes cut across multiple domains of context, including CFIR’s domains of the innovation, outer setting, inner setting, individuals, and implementation process, and often contained both barriers and enablers to implementation, reflecting the complexity of delivering this type of care. An overview of these themes is provided in Table 3.

**Table 3:** Overview of key implementation themes identified by parents and providers in the qualitative evaluation of CHD LIFE care pathway

Implementation Themes	Description
Holistic, evidence-based, and co-designed care^a^	CHD LIFE’s patient-centered, evidence-based, multidisciplinary and holistic approach is a significant strength.
Centralized foundations of pathway engagement	Having a centralized education and referral process at the tertiary center is important for successful pathway implementation and was generally working well. However, concerns were raised about the timing of parental education to best support improvements in knowledge and awareness.
Decentralized follow-up: a double-edged sword	There are both benefits and challenges to providing decentralized follow-up care in community. Despite challenges within the service delivery system, decentralized care was favoured by families and professionals.
Pathway flexibility is a strength, but needs implementation support	While the CHD LIFE pathway’s focus on principles and flexibility supports regional intervention tailoring, a lack of a formalized structure and support sometimes created confusion for both providers and families regarding pathway requirements.
Inconsistent pathway implementation and awareness	Variable effectiveness of implementation has been noted across the state and within services, leading to inconsistent provider awareness and mixed experiences for families seeking care.
Integrating and aligning care with existing systems and services	The pathway works well integrated into the existing model of childhood follow-up care and leveraging services in community. While CHD LIFE aligned well with child health nursing roles, the role of GPs in the pathway is currently limited and unclear.
Sustaining engagement and follow-up within the system	The CHD LIFE care pathway was perceived as a “safety net”, providing structure and reassurance for families through regular prompts and check-ins. However, its implementation was hindered by the lack of an ongoing touchpoint to support parents and providers. Physical reminders, such as stickers and postcards, demonstrated mixed effectiveness.
Key role for families in driving care	The empowering benefits of the CHD LIFE pathway as a parent-centered model of care were outweighed by the demands placed on families to navigate services and maintain engagement with the pathway. For some, a greater sense of partnership was needed.
Importance of caregiver characteristics	Due to its parent-centeredness, many stakeholders believed that the pathway’s success was partly reliant on each caregiver’s individual characteristics, background and circumstances. This may create disparities in care and effectiveness.

^a^theme only reported by service providers.

#### Holistic, evidence-based, and co-designed follow-up care

Providers emphasized that CHD LIFE’s holistic, multidisciplinary approach, grounded in evidence and co-designed with families, is a significant strength. It was seen to incorporate best practices in cardiac neurodevelopment with broader developmental theory, supporting a focus on children’s functioning within their family and community environment. As one provider stated:

*“Optimising developmental outcomes is more than accessing healthcare services. It’s also about the wrap around services that exist within people’s communities, how the school operates, how informal supports operate.”* [P42, manager].

#### Centralized foundations of pathway engagement

Although the CHD LIFE care pathway is decentralized in delivery, stakeholders emphasized that key processes at the tertiary center—such as identification, onward referral, and education of children and their families—were critical foundations for successful pathway implementation. This step ensures families are introduced to the pathway, receive information about developmental risk and concerns, and are appropriately linked to local follow-up services. Providers highlighted the value of dedicated staff, good teamwork and communication, leadership buy-in and champions, and ongoing training to support these processes.

Parents spoke positively about the tertiary center and its staff, recalled receiving pathway information and materials, and valued education about potential developmental concerns. One parent noted: *“They were really good about explaining CHD LIFE to us. So, we were given a heads up about it [development] at that point in time which was actually really helpful.”* [F14, parent]. However, others found the information difficult to process, particularly when it was delivered before hospital discharge and struggled to articulate what the pathway involved: *“To be honest I don’t know what the CHD LIFE program sort of encompasses”* [F19, parent]. The overwhelming nature of this period often impacted parents’ ability to engage fully with the material: *“It was definitely discussed when I signed up to this. I was trying to remember everything, it was all a bit of a blur but it was definitely discussed.”* [F01, parent]. Many families and professionals noted the importance of revisiting this education at a less overwhelming time.

#### Decentralized follow-up: a double-edged sword

How the decentralized nature of follow-up care impacted implementation was frequently discussed by both professionals and parents. While the decentralized model was broadly perceived to offer the best fit for Queensland’s context and be acceptable to both families and providers, it was also acknowledged to come with trade-offs that could challenge effective implementation. Two sub-themes captured this inherent tension. Decentralized care was perceived to be a strength (sub-theme one with six codes, Table 4) due to its geographical fit, reduced resourcing requirements, early engagement with local services, reduced burden on families, greater convenience of access, and avoidance of hospital-related trauma. However, participants also identified significant challenges with decentralization that limited the pathway’s effectiveness in some settings (sub-theme two with five codes, Table 5). These were largely related to contextual factors beyond the control of the CHD LIFE pathway team, including local service availability for evaluation and intervention (e.g., long waitlists, lack of specialists), fragmentation of communication, and perceived gaps in the skills and knowledge of community-based providers. These challenges were not seen as a failure of the model itself, but rather reflections of broader resourcing and infrastructure gaps in local contexts.

**Table 4:** Reported strengths of decentralized neurodevelopmental follow-up care via the CHD LIFE pathway in Queensland

Strengths	Description	Supportive quotes
Geographically appropriate model (SP)	Decentralized care was perceived to be a good fit with the geography of Queensland, with a relatively small cohort of children dispersed across a large geographical area. Decentralizing care was seen as more equitable than centralizing care to provide follow-up for all children across the state.	*“I think with the state, the size it is and … kids come from everywhere, it makes much more sense to me to have it decentralized, and they’re seeing their own local services.”* [allied health provider] *“The thought was it would be more equitable across the board and more sustainable in being locally driven.”* [allied health provider]
Reduced resourcing requirements (SP)	Previous centralized versions of follow-up at the tertiary center had proved unsustainable, particularly for resourcing allied health provider involvement. As CHD LIFE’s decentralized follow-up care pathway mostly utilizes existing funded services, it is less costly to deliver, improving its chances of sustainability and extending eligibility to a broader cohort.	*“Resources in neurodevelopment are very limited. So, it’s very hard to access funding and resources to provide assessments. So, we were left with utilising the best approach to put something sustainable in with resources that are already available within the health community, within the state.”* [cardiologist]
Strengthens engagement with local services (SP)	There are skilled developmental providers and services in the community. The CHD LIFE pathway taps into their expertise and knowledge to optimize care and function of children in their local environment, including community services to meet broader needs outside of screening and assessment. Handing over care to local services early on helps to build trust and continuity for families and to address the assumption that the best care is at the hospital.	“*These issues are things that they’re seeing all the time and treating all the time for a whole range of different children.”* [hospital-based manager] *“It’s about supporting families to have the confidence to continue to link back into their local communities because it’s natural for families to think you’re experts in congenital heart disease, so we’ll keep speaking to you and we’ll cut out our local people. The reality is, to deliver good developmental services, you don’t need experts in congenital heart disease, you need experts in child development.”* [hospital-based manager]
Less travel and time burden for families (SP and F)	Families reported that not having to travel to access follow-up care meant the process was less burdensome. They appreciated having fewer disruptions, incurring less expense, and spending less time away from work.	*“We’re so much happier to stay in [regional city]. It’s really hard to travel with my daughter, she never stops. I wouldn’t want to be having to get back on a plane and going back to Brisbane.”* [F11, parent from regional area]
Greater convenience of access (SP and F)	Having follow-up care locally in the community was reported to be much more convenient for families, including things such as physical access and greater flexibility in scheduling appointments.	*“I prefer the community just because it was so much easier to get to than going to the hospital. We can also pick and choose [appointments] and we don’t need to worry about the hassle of getting to the hospital.”* [F16, parent] *“It gave more access to families, so families didn’t have to travel down to Brisbane to access neurodevelopmental follow up. They could access that in their local community. It was available.”* [community provider]
Avoidance of hospital-related trauma (SP and F)	Care in the community avoids exacerbation of any trauma parents or children may feel upon returning to the tertiary hospital for follow-up.	*“I didn’t want to go back to Queensland Children’s Hospital. Too much trauma surrounding that time for me, lots of PTSD. I just never wanted to go back there if I didn’t have to.”* [F17, parent]

*SP* reported by service providers, *F* reported by families.

**Table 5:** Reported challenges of implementing decentralized neurodevelopmental follow-up care via the CHD LIFE pathway in Queensland

Challenge	Description	Supportive quotes
Inconsistent availability and access to local developmental services (SP, F)	While screening services were readily accessible to families, oftentimes long waitlists, limited availability, and high costs of developmental services and providers in the community limited timely access to evaluation and intervention services. This impacted the effectiveness of the CHD LIFE care pathway beyond screening for some families.	*“So, once you get outside of primary care, there’s enormous wait lists. If we’re doing all this great screening and picking up things, they’ve still got to get into a system to be more fully assessed and then ideally to have intervention…that’s potentially a barrier.”* [hospital-based manager] *“They’ve successfully been on the pathway, found to have a problem, but they’re waiting nine months for a child development service appointment. So the pathway is working in that sense, but it doesn’t seem to cut through the public health issue with wait times.”* [cardiologist] *“There was such a big waitlist through the Child Development Service…it’ll take about six months to see someone, and he’ll only get six sessions.”* [F01, parent] *“We spent $5 000 on private sessions. We can afford to but for low-income families it would be very difficult.”* [F15, parent]
Variable expertise and confidence in local providers (SP, F)	Both health providers and parents pointed out the greater variability in skills and knowledge of those providing developmental follow-up in the community compared to a small team at a centralized program. Some parents perceived health providers working in the community to have less specialist cardiac developmental knowledge than those at tertiary hospital centers, which impacted their trust in using them for follow-up. Moreover, some parents perceived care in community to be of a lesser standard than that available at the hospital.	*“You’re going to be assessed by different people with different training levels and different skill sets. And do you get the same quality of assessment in each centre? I don’t know?”* [cardiologist] *“When they’re community based, they’re not always as knowledgeable specifically with the CHD stuff that we deal with.”* [F04, parent] *“I’m not in Brisbane getting the reviews, am I getting the same level of care? But I still trust the Child Development Team locally so it’s just weighing it out in your head.”* [F13, parent in regional area]
Variable local resourcing (SP, F)	With follow-up delivered locally, pathway success depended on the resources available within the local community to provide care. This was not equitable across the state. A lack of services or specialists in regional areas (GPs, allied health, pediatricians), high staff turnover and providers who did not meet families’ needs were reported as key challenges in some regions.	*“But I also think it’s quite hard when a family gets on the pathway, they might go back to their regional community and then there’s nothing there for them, so then it’s really hard for them to stay on that pathway.”* [external manager]
Fragmented communication and patient tracking (SP)	Decentralization of care across the state and different services meant that there was little line of sight to other parts of the pathway or what happened to families once referred. Communication around this can be lacking, and data systems don’t always link up into primary care making it hard to track children through the follow-up journey.	*“What I’ve always been worried about is when we discharge them, just making sure families stay on that screening pathway because we have no line of sight to that.”* [hospital allied-health provider] *“And then sending someone …and hoping that the parent and the community child health clinics have enough awareness to do those tests and screening processes.”* [hospital-based manager]
Focus on screening (SP)	The CHD LIFE pathway has a large focus on screening (as opposed to assessment as minimum care standard), which was perceived to lack the thoroughness which could be provided in a centralized model.	*“We did the centralized model…it was more service than they’re currently getting through that pathway, but it just wasn’t doable. In the ideal world, it would be a little bit more thorough, as an assessment rather than just screening, but I think this is the best we can do.”* [allied health provider]

*SP* reported by service providers, *F* reported by families.

#### Pathway flexibility is a strength, but it needs implementation support

The CHD LIFE care pathway is designed around guiding principles rather than a rigid protocol, allowing contextual adaptation across the state: “*it might look different in different parts*” [P31, health manager]. Stakeholders viewed this flexibility as a strength, encouraging local health service ownership and buy-in, and being responsive to family needs. However, successful regional adoption requires substantial local engagement and capacity building, which has been inconsistent across Queensland’s 16 public health service jurisdictions. Without a formalized structure to support local tailoring and ongoing implementation, confusion for both providers and families often occurred regarding pathway requirements or components.

#### Inconsistent pathway implementation and awareness

While the pathway’s flexible design was appreciated, both health professionals and parents reported this often meant that translation into practice was uneven, resulting in inconsistent awareness, service delivery, and experiences when seeking care locally. Eleven parents reported negative experiences, including encounters with providers unfamiliar with the care pathway: *“Every time we turned up [to child health service], the person didn’t know what I was talking about”* [F21, parent]. Some parents also noted a lack of CHD-specific developmental knowledge among providers: “*We were saying we know she’s got a high risk of having issues, and you should too*” [F05, parent]. In contrast, other parents—particularly those whose children had fewer developmental concerns—shared positive experiences: “*it was just such a core component of our journey”* [F13, parent].

This inconsistency was particularly marked among general practitioners (GPs), with providers noting that GPs had limited knowledge of the care pathway, and families reported mixed experiences of seeking neurodevelopmental follow-up care with their GP. While some families reported positive engagement, (“*I think his GP has been our best resource”* [F16, parent]), others described struggling to convince their GPs of the importance of developmental follow-up: *“It was almost like we had to get them [GP] to believe us that something was going on…fight to be believed”* [F05, parent].

#### Integrating and aligning care with existing systems and services

Health professionals emphasized that the CHD LIFE pathway works well integrated into the existing childhood developmental follow-up care model in Queensland. This includes leveraging community-based services such as child health nurses, allied health providers, and pediatricians. Child health nurses, in particular, described the care pathway as aligning with their roles in screening, education, and holistic support. *“It’s part of our core business… to assess children’s growth and development and provide early intervention strategies… It really does fit nicely with the child health nurse role”* [P66, community nurse]. This alignment was seen to promote sustainability through reduced reliance on centralized tertiary services.

In contrast, the role of GPs within the pathway was less clear. Stakeholders highlighted variability in GPs’ skills and interest in child neurodevelopmental care, with parents often having to “shop around” for the right fit. Despite these challenges, families and providers agreed that GPs play an important role in supporting continuity of care and should be more systematically included in the care pathway.

#### Sustaining engagement and follow-up within the system

Despite its decentralized nature, the CHD LIFE pathway was widely perceived as offering a “*safety net*” [multiple parents and providers] for families through scheduled follow-up and the reassurance of regular check-ins with health professionals. Parents appreciated the sense of ongoing support and felt less isolated in their follow-up journey. As one parent explained: *“I think for me, it’s that he’s not forgotten. That he still exists. Like there’s still people who care”* [F01, parent]. Similarly, providers felt reassured that families would not be left without support after discharge from tertiary care. One allied health professional noted: *“…these kids have got that safety net … they’re getting that checked up upon…that if something goes wrong that they’ll be picked up and managed.”* [P68, allied health provider]. However, this safety net was not always reliable. Both parents and providers described instances where families did not or could not access recommended developmental follow-up care, only to reengage later, after concerns had been identified.

A related challenge for families in sustaining community-based follow-up was the absence of a consistent touchpoint after leaving the tertiary center. Many expressed the need for a care navigator, “help desk,” resource, or contact person to provide ongoing pathway support and navigation advice, or milestone reminders: *“No one’s contacted us and gone you’ve got an appointment coming up … so we are just floating.”* [F02, parent]. This presented a barrier for parents and providers to access timely information about developmental milestones, or pathway and service requirements, especially as children “aged-out” of child health services (typically at 5 years old). As a result, some families felt unsupported and at risk of being overlooked: *“I feel like you’re kind of just left to your own devices and that’s so [expletive] scary, it’s so scary.”* [F18, parent]. In contrast, families who received reminders through local services or were part of the original centralized program felt more supported: “*Sometimes we wondered if we’d be forgotten, but we never were.”* [F23, parent, centralized program].

Physical prompts, such as stickers and postcards, added to the existing child health record were highlighted as important enablers for implementation by ensuring visibility of children on the care pathway within the existing childhood follow-up system. Parents agreed that these flags prompted them to seek regular screening for their child. However, these flags were less effective at enabling implementation by providers. Many parents described needing to “*nag*” [multiple parents] or prompt child health nurses or GPs to perform the pathway-specific Ages and Stages Questionnaire rather than defaulting to standard screening checks: *“Even our Red Book has a sticker on it saying we need to have the Ages and Stages done but it seems like a lot of the child health nurses aren’t aware of that requirement.”* [F11, parent].

#### Key role for families in driving care

In the CHD LIFE care pathway, families were reported to play a central role in driving follow-up, including remembering milestones, booking appointments, and finding relevant services and supports after initial referral: *“this is driven 100% by us, that’s the feeling that I have”* [F01, parent]. Both parents and professionals agreed this approach allows for autonomy and flexibility, enabling families to make decisions tailored to their needs: *“It’s okay for families to say, well, I would rather do this versus that. So, I don’t want to go to child health and have that screening, I want to go to my GP.”* [P31, hospital-based provider]. At the same time, families also valued that the pathway was designed as a partnership with care providers, supplementing caregiver intuition and experience with professional reassurance. As one parent described: *“Validation that a lot of the things that I was concerned about were sort of delayed and were something that needed to be worked on. And like being taken seriously about my concerns”* [F04, parent]. Additionally, the pathway was reported to empower families to engage in follow-up care, fostering a strong sense of advocacy for their children.

*“Parents [are] feeling an assurance that neurodevelopmental follow up is important, and they’ve got a role to play in that…stories are changing in how families approach the importance of neurodevelopment.”* [P67, community allied health provider].

Despite these positives, many families reported feeling overwhelmed by the responsibility of driving care, and that a stronger sense of partnership was still required. The overwhelming feedback from parents is that this type of follow-up care model places substantial demands on families, which can affect their ability to maintain consistent and sustained engagement in the care pathway: “*if we weren’t driving it, you could actually fall through the cracks*” [F12, parent]. Providers also noted that engagement often diminished as caring demands increased or life circumstances changed: *“You can see in the notes how families start off keen, and then …life gets in the way, and sometimes they lose sight of the importance of those checks”* [P58, hospital-based manager]. Many parents reported challenges navigating a complex and fragmented system independently without clear guidance on eligibility, service availability, or regional variations: “*I didn’t really know where to start, so it was backwards and forwards all the time and I didn’t really know what to do.”* [F24, parent].

#### Importance of caregiver characteristics

Due to its parent-centeredness, many stakeholders believed that the pathway’s success was partly reliant on each caregiver’s individual characteristics, background, and circumstances. Factors such as health literacy, confidence to ask questions, education, parenting style, demographics, financial resources, and prior engagement with health services shaped their ability to follow the pathway. One parent highlighted this disparity: *“I do feel for families who perhaps aren’t as comfortable in a medical situation, where perhaps English isn’t their first language, or they’re not as familiar with medical terms or scientific terms…perhaps some of those children are then slipping through the gaps more than they should be.”* [P11, manager].

### Perceived impacts of the CHD LIFE care pathway

In addition to examining the processes of implementation, this study also explored stakeholders’ perceptions of the CHD LIFE care pathway’s effectiveness. Analysis revealed a range of perceived impacts of the pathway on providers, parents, and children which could be grouped into five distinct themes (Table 6). These included acceptability of the pathway by parents and providers; increased developmental awareness and capacity building for providers and parents; and increased parental confidence to be advocates for their child. Parents also reported more timely access for children to assessment and intervention, with many attributing observed improvements in their child’s development to being able to access pathway supports. Although not considered a main impact theme, several service providers also described broader system-level impacts as a result of embedding the pathway into the existing pediatric developmental care system in Queensland. For example, more systematic use of the Ages and Stages screening tool was integrated into child health services.

**Table 6:** Overview of perceived impact themes identified by parents and providers in the qualitative evaluation of the CHD LIFE care pathway

Impact theme	Impact sub-theme	Description	Supportive quotes
Pathway acceptability (SP, F)		Despite noted challenges and variable implementation success, both parents and providers expressed high levels of satisfaction and positive, rewarding experiences with the CHD LIFE care pathway overall.	*“Overall, it’s been a really positive experience for us”* [F20, parent] *“It’s something I’m very well aware of and I feel very, very strongly in favour of.”* [allied health provider]
Increased knowledge, awareness, and skills for developmental follow-up (SP, F)	Increased awareness and capacity building of service providers	The CHD LIFE program and care pathway were noted to build awareness and capacity for developmental follow-up in child health nurses, cardiologists, and GPs. This included increased understanding about the importance of follow-up for children with CHD, acknowledgment of their own role in prompting or supporting families on the pathway, using Ages and Stages to screen children, and referring families to appropriate services.	*“That’s educated me more as well. So, I’m really confident to be able to talk to a family about, you know, how we can access these services, why it’s important.”* [non-health provider] *“You can see in things that they’re [providers] documenting that there is very much an awareness of the pathway.”* [cardiologist] *“And I think that’s been the other benefit …educating the cardiologists as well. We’ll get referrals from the cardiologists because of the joint work and the education”* [child development services provider]
	Increased parental knowledge and awareness	The CHD LIFE care pathway has increased parents’ knowledge and awareness about monitoring developmental milestones and raising concerns for their child with CHD. Even parents with prior knowledge about childhood development found the contextualization of this knowledge to their child with CHD was beneficial.	*“It’s made a big difference. I didn’t know there would be any neurodevelopmental delays or anything until the lady spoke to me about it at the hospital”* [F01, parent] *“The pathway is definitely providing that information. Families that we’ve heard from that have been on the pathway, they’re aware that they need to act on these things.”* [other heath provider]
Increased parental confidence to be the child’s advocate (SP, F)		The CHD LIFE care pathway enabled parents to take a more proactive approach to their child’s development, including prioritizing check-ups and engaging with primary care and other developmental activities such as playgroups, gymnastics, and home play with their child. Parents reported that they felt better equipped and more confident to advocate with healthcare professionals for referrals to assessment or interventions for their child, even if providers dismissed their initial concerns.	*“I wouldn’t have been as aware of developmental problems. So maybe I would have just rolled with it a bit more rather than being as full on trying to access everything we could.”* [F03, parent] *“We just kept constantly bringing it up as many times as we could [developmental concerns]. And for everyone we saw we brought it up.”* [F05, parent] *“We do see a lot more families accessing or trying to access support services… a lot more families coming to us asking for assistance in how to access those services.”* [other health provider]
Facilitates timely access to diagnosis and intervention (F)		Parents believed that being on the CHD LIFE pathway resulted in more timely identification of developmental concerns for their children than would otherwise occur. They also reported that receiving a diagnosis via the pathway allowed them to access early intervention services at the level required to meet the needs of their child. For some children, the CHD LIFE pathway also helped to facilitate access to supports via the National Disability Insurance Scheme.	*“I think we would have been on a very slow boat. I think we would be way behind if we hadn’t been plugged in and got the things we did”* [F08, parent] *“Me being early (to get assessment) definitely helped him. If I hadn’t have got that he would be well behind, he wouldn’t have been reaching his potential now.”* [F06, parent]
Developmental improvements observed in children (F)		Twelve parents mentioned developmental improvements in their child after accessing services such as speech therapy, physiotherapy, exercise physiology, occupational therapy or parental support classes through the CHD LIFE care pathway.	*“I would say it’s been lifesaving for [child]. He’s just thriving now. And I know for sure that if we didn’t have that early intervention, he wouldn’t have the outcomes he has now.”* [F04, parent] *“Exercise physiology has been wonderful for her…It helped her catch up a bit”* [F09, parent]

*SP* reported by service providers, *F* reported by families.

### Opportunities to improve the CHD LIFE care pathway

Stakeholder-informed suggestions for improvement encompassed six thematic areas, which aligned closely with the barriers and enablers identified in the implementation summary domain, reinforcing their relevance as context-specific strategies. Suggested improvements include ability to access an identified contact, touchpoint or resource to support follow-up of families where this can’t be found locally; family check-ins or brief follow-up with tertiary staff after hospital discharge; awareness and capacity building of parents and providers; greater structure and clarity about the care pathway for parents and providers; improved communication, engagement and coordination between specialists and local services/hospitals; and mental health and well-being support for parents. These improvement themes are explained in greater detail in Additional File 4, Supplementary Tables S2–S7, including the rationale for improvement, possible forms it may take, and potential benefits, accompanied by illustrative quotes.

## Discussion

This qualitative process evaluation of a decentralized model for neurodevelopmental follow-up of children with CHD provided favorable findings regarding its implementation and perceived impacts. Both families and healthcare providers reported positive impacts, with outcomes such as acceptability, improved access, and capacity building aligning with findings from evaluations of centralized follow-up programs.^19–21^ However, by embedding follow-up care into primary health and community settings, CHD LIFE offered several advantages. It reduced geographic, logistical, and cost barriers for families while leveraging local resources, fostering capacity building in community services, and empowering families in their child’s care. These findings highlight how decentralized models may address common limitations with centralized follow-up care, particularly in geographically diverse regions. Yet, challenges related to provider knowledge and engagement with the care pathway, variable regional implementation, and the heavy reliance on local service availability and family engagement underscore the ongoing need for targeted strategies to support local providers, standardize implementation processes, and reduce the burden on families.

### A contextually tailored decentralized model of care

The CHD LIFE care pathway represents a unique approach to addressing the neurodevelopmental needs of children with CHD in a way that is responsive to the local Australian context. In contrast to the hospital-based programs common in North America, the CHD LIFE model embeds care into local primary health and community settings. This decentralized approach reflects the geographical and infrastructural realities of Australian care delivery, where many families live hundreds of kilometers from tertiary cardiac services but much closer to primary care services.^22^ The pathway also capitalized on existing system enablers, including a state-wide developmental surveillance process, well-established child health and development services, a strong state-wide network of health professionals, and universal health coverage. The pathway’s consideration of local context underscores the value of tailoring care models to regional infrastructure and need, with CHD LIFE representing a replicable yet adaptable framework for other settings with similarly dispersed populations and robust local infrastructure.

CHD LIFE’s decentralized pathway also aligns with calls for innovative approaches to address barriers in traditional centralized care models.^6,11^ However, it is just one approach with a range of other decentralized models possible, including the use of telehealth,^23^ postal-based screening,^24^ centralized support of local clinics,^25^ phone calls,^20^ outreach, or delivery in schools.^26^ The optimal approach, or combination of approaches, is likely to depend on the local context, available resources, and the needs of the target population. While evidence suggests that the effectiveness of decentralized models in healthcare is highly dependent on the implementation context,^27^ evaluations of such models in neurodevelopmental follow-up care and the contexts that support their use are scarce. Consequently, future research should prioritize understanding the contextual factors that may influence follow-up model design and hinder or assist implementation in particular settings. This may be particularly beneficial when considering implementation of neurodevelopmental and neurocognitive care recommendations from the recently established Australian National Standards of Care for Childhood-onset Heart Disease.^5^

While decentralization enables local access, our findings underscore the ongoing importance of centralized governance, support, and structure for effective implementation of these models of care. Initial education and referral provided at the tertiary center were critical enablers of family engagement with the CHD LIFE pathway. From a health system perspective, having a centrally based clinical team, leadership group, and governance of the pathway was important. However, given that both families and providers reported needing guidance and support from the tertiary center beyond what local resources could offer, enhancing centralized support (e.g., web page, staff, or resources) could strengthen the pathway’s decentralized implementation. Reviewing and refining the balance between centralized support and localized execution, as recommended in the Australian National Standards of Care for Childhood-onset Heart Disease,^5^ will help to ensure that both providers and families are equipped to support neurodevelopmental needs effectively.

CHD LIFE emphasizes universal developmental screening using the Ages and Stages Questionnaire-3 (ASQ-3), followed by targeted pathways for children requiring further evaluation and intervention. This stepped approach contrasts with the largely evaluation-focused follow-up practices recommended in US-developed guidance.^6^ However, challenges in implementing this guidance outside of urban US contexts have prompted calls for adaptation to other countries and contexts.^28,29^ Our evaluation demonstrates that a tiered strategy can identify children who may need more intensive neurodevelopmental support, aligning with emerging neuropsychology evidence.^30,31^ It also addresses parental concerns about the stress of attending repeated formal evaluations early in a child’s life.^32^

### System-level implications for implementation

The CHD LIFE care pathway illustrates how neurodevelopmental follow-up can be integrated into existing health systems by local resources. This is important given the persistent resourcing constraints reported for standalone CHD follow-up programs in Australia^9,33^ and internationally.^20^ In the US, a recent analysis of claims data also suggests that much neurodevelopmental care for children with complex CHD, including developmental and psychological testing, already occurs in community-based services.^34^ Nevertheless, absolute follow-up rates remain low across both formalized center-based programs and visits to community services in North America, with estimates at 25-30%.^34,35^ This highlights the potential for programs like CHD LIFE to supplement centralized care models internationally.

The success of such an approach, however, depends heavily on the availability of broader system resources, which can vary by location. For example, integrating the CHD LIFE care pathway into an already overburdened healthcare system presented challenges, particularly in regional areas. Although screening was generally accessible, families frequently reported prolonged wait times for developmental assessments due to service capacity and staffing constraints of existing services. This is not unique to CHD LIFE, but reflects a system-level issue previously reported in Australian and American studies of access to specialist developmental services.^36,37^ Investing in workforce development, integrating telehealth services, advocating for adequate funding, optimizing resource allocation, and streamlining care pathways may be important steps toward more effective integration of the CHD LIFE care pathway in the Queensland healthcare system.

A further system-level challenge for CHD LIFE was maintaining a consistent standard of care across the state while decentralizing care delivery into local services. Although early and ongoing strategies supported state-wide adoption, these efforts were limited by the resources available at the cardiac surgical center, with few formal mechanisms for sustained pathway implementation support. While variability in care has also been noted in hospital-based follow-up programs,^38^ it is often more likely in decentralized models where disparities in local governance, institutional capacity, engagement, and resource allocation can result in inconsistent health service delivery.^39^ While it can be difficult to mitigate these challenges completely, it is important to consider strategies that could help alleviate some of the implementation variability. In particular, the findings underscore the need to address ongoing gaps in provider awareness and engagement in particular regions or clinical groups, especially for those who have not been targeted with educational interventions to date (e.g., GPs). Targeted and readily accessible education for all providers, along with additional practical resources, is essential if they are to effectively support families to navigate care.

### Implementation implications for families

The CHD LIFE care pathway represents a neurodevelopmental follow-up model for children with CHD that aligns with guidelines advocating for patient- and family-centered care. This is particularly significant, as family-centered care can improve social, psychological, and quality-of-life outcomes for children and families, increase satisfaction with services, and empower caregivers through skill and capacity building.^40,41^ Findings from the evaluation suggest that the CHD LIFE pathway exemplifies these benefits by fostering greater awareness of developmental milestones and enhancing parental advocacy. However, it also highlights challenges for families tasked with leading care without sufficient ongoing support. This challenge is not unique to the CHD LIFE pathway, with similar concerns identified from studies originating in Canada and Switzerland.^32,42^ Finding the right balance between parent-led and provider-supported follow-up is important to avoid overburdening families already experiencing the stress associated with managing a child with CHD.^43^ Additionally, these findings reinforce the need to include screening for parental mental health and access to psychological and social supports for caregivers and children in follow-up care.^2^

Our findings also underscore the critical need for structured support systems that complement parent-led follow-up models, both at tertiary centers and in local communities. This is especially important for families from socioeconomically disadvantaged or culturally marginalized backgrounds who may face greater challenges navigating complex care. Introducing or expanding access to ongoing supports, such as care navigators or formal contact points, could help reduce parental burden and improve engagement with the pathway.^44^ Service-initiated check-ins and reminders at key developmental time points could further enhance support, as they have done in hospital-based programs.^35,45^ Additionally, addressing parents’ desire for improved access to timely, trusted, and tailored information about CHD, neurodevelopment, and the care pathway is crucial and a need shared by families across various international contexts, including those in centralized follow-up models.^46,47^

### Strengths and limitations

A major strength of this study is its comprehensive qualitative approach, capturing multiple viewpoints, triangulation of data across groups, and the presence of both positive and negative cases (deviant cases) to enhance the credibility and applicability of the findings. Additionally, the study’s combined focus on parent perspectives, decentralized care, and process evaluation addresses the critical need for research exploring the feasibility, acceptability, and experiences of different processes and models of care in neurodevelopmental follow-up.^7,11^ Limitations include potential underrepresentation of participants from some non-metropolitan regions, the predominance of highly educated families among participants, and the limited amount of data describing GP involvement. Finally, while study participants reported positive impacts perceived to be associated with the CHD LIFE pathway, in the absence of controlled testing, these need to be interpreted with caution.

## Supplementary information

Supplementary Information

## Data Availability

Data are not publicly available due to information that could compromise the privacy of research participants if published. However, excerpts of the transcripts relevant to the study that support the findings are available on request from the corresponding author.
