# Supplementary material for: Family and health professional perspectives of the CHD LIFE cardiac developmental long-term care pathway: a qualitative evaluation
Source: Pediatr Res. 2025 Sep 30;99(6):2229–41. doi: 10.1038/s41390-025-04380-8 (PMC13433239; doi:10.1038/s41390-025-04380-8)
Supplement: Supplementary file 1 [file 41390_2025_4380_MOESM1_ESM.pdf]

# Additional file 1: Completed consolidated criteria for reporting qualitative studies (COREQ) checklist

**Supplemental Table S1**. Completed COREQ 32-item checklist for transparent and complete reporting of methods

| COREQ checklist | Location where item is reported |
|---|---|
| **Domain 1: Research team and reflexivity** | |
| *Personal Characteristics* | |
| 1. Interviewer/facilitator | Methods- Data collection |
| 2. Credentials | Methods- Data collection |
| 3. Occupation | Methods- Data collection |
| 4. Gender | Methods- Data collection |
| 5. Experience and training | Methods- Data collection |
| *Relationship with participants* | |
| 6. Relationship established | Methods- Data collection |
| 7. Participant knowledge of the interviewer | Methods- Data collection |
| 8. Interviewer characteristics | Methods- Data collection |
| **Domain 2: study design** | |
| *Theoretical framework* | |
| 9. Methodological orientation and Theory | Methods- Study design |
| *Participant selection* | |

| COREQ checklist | Location where item is reported |
|---|---|
| 10. Sampling | Methods- Study participants and recruitment |
| 11. Method of approach | Methods- Study participants and recruitment |
| 12. Sample size | Methods- Study participants and recruitment |
| 13. Non-participation | Results- Participant characteristics |
| *Setting* | |
| 14. Setting of data collection | Methods- Data collection |
| 15. Presence of non-participants | Methods- Data collection |
| 16. Description of sample | Methods- Study participants and recruitment |
| *Data collection* | |
| 17. Interview guide | Methods- Data collection |
| 18. Repeat interviews | Methods- Data collection |
| 19. Audio/visual recording | Methods- Data collection |
| 20. Field notes | Methods- Data collection |
| 21. Duration | Methods- Data collection |
| 22. Data saturation | Methods- Data collection |
| 23. Transcripts returned | |
| **Domain 3: analysis and findings** | |
| *Data analysis* | |
| 24. Number of data coders | Methods- Data analysis |

| COREQ checklist | Location where item is reported |
|---|---|
| 25. Description of the coding tree | Methods- Data analysis |
| 26. Derivation of themes | Methods- Data analysis |
| 27. Software | Methods- Data analysis |
| 28. Participant checking | Methods- Data analysis |
| *Reporting* | |
| 29. Quotations presented | Results |
| 30. Data and findings consistent | Results |
| 31. Clarity of major themes | Results and Table 3 |
| 32. Clarity of minor themes | Results and Table 3 |

Evaluation of CHD LIFE Neurodevelopmental Long-term Follow up Program (CHD LIFE)
**QUT Ethics Approval Number xxxx**

# Semi-Structured Interviews with CHD LIFE families and carers: Outline

**Aims:**

- Highlight contextualised barriers and facilitators to the implementation and delivery of the CHD LIFE cardiac developmental long-term care pathway and neurodevelopmental follow-up care throughout Queensland
- Assess implementation outcomes such as change in reach of services, adoption of program activities by families, and perceived fit of service with family needs
- Assess relevant service outcomes such as increased timeliness or access to care, and onward referrals to other services
- Assess child and family outcomes such changes in knowledge, costs, travel, acceptability of care, services used, quality of life.

**Structure:**
The interviews will be semi-structured, with open-ended questions which are adaptable to reflect the responses being provided. The conversation should explore issues as they are raised, with guidance and prompting as required. Interviewers/facilitators will ensure discussion progresses in a timely, yet informative manner. Interviews will last no more than 40 minutes via Zoom, Teams, phone, or in-person.

**Participants:**
Interviews will be held with the parents/carers of children who have been referred to services via the CHD LIFE care pathway and consented to the CHD LIFE Program database. Potential participants will be recruited via an email, SMS or phone invitation sent to families who have consented for future research.

**Process/Content:**
Review aims of interview and participant information sheet, particularly confidentiality, consent, privacy and right not to participate. Check and collect signed consent form or e-consent and begin recording or note-taking.

The content of the interviews is largely based on (a) capturing established implementation and health service outcomes and (b) examination of barriers and enablers underpinned the Consolidated Framework for Implementation Research1,2. CFIR is a widely cited and rigorously developed implementation determinants framework that was developed through a process of consolidating earlier published literature. It has a focus on how the inner setting, outer setting, intervention characteristics, individuals, and implementation processes influence implementation outcomes such as reach, appropriateness and effectiveness.

Two questions are also derived from The Community Services Outcomes Tree framework for capturing the outcomes that individual's experience as a result of community services[3].

**References**

1. Damschroder LJ, Aron DC, Keith RE, Kirsh SR, Alexander JA, Lowery JC. Fostering implementation of health services research findings into practice: a consolidated framework for advancing implementation science. Implementation science. 2009 Dec;4(1):1-5.
2. Damschroder LJ, Reardon CM, Widerquist MA, Lowery JC. The Updated Consolidated Framework for Implementation Research: CFIR 2.0. Research Square (preprint). 2022. DOI: https://doi.org/10.21203/rs.3.rs-1581880/v1
3. Wilson, E.; Campain, R.; Brown, C. (2021). Community Services Outcomes Tree. https://communityservicesoutcomestree.com

# Semi-Structured Interviews with CHD LIFE families: Proposed questions

**Before beginning the interview**

| **Location/Setting** | **Y/N** | **Notes** |
|---|---|---|
| *Is the location private?* | | |
| *Is the location neutral for all participants?* | | |
| *Have accessibility considerations been addressed?* | | |
| *Have all WHS procedures been addressed?* | | |
| *Are there any other considerations to be noted for records prior to conducting the interview/focus group?* | | |
| *In-person COVID-19 precautions*<br>• *Confirmation of self-reported COVID-19 status (no symptoms, not awaiting test, not required to isolate or self-quarantine)?*<br>• *Addressed current government mandated restrictions in location of interview?*<br>• *Appropriate ventilation (e.g., windows open)?*<br>• *Hand hygiene amenities available?*<br>• *Space for appropriate social distancing?*<br>• *Masks if required/wanted?* | | |

**Introduction**

Hi, my name is [_______]. I'm speaking with you today because we are interested in your thoughts about the neurodevelopmental follow-up care for children with congenital heart disease, and specifically the CHD LIFE Program. The information you provide to us will be used to help evaluate the CHD LIFE cardiac developmental long-term care pathway and to improve how it is delivered across the state.

There are no right or wrong answers and any information you can provide will be helpful. Don't worry if you feel you cannot respond to some questions. It is fine to let me know you are unsure or do not know.

We will record the conversation with you if that is OK and will only share your conversation with other members of the research team or by making sure other people cannot identify your responses. We take your privacy and confidentiality seriously. We don't need you to use any specific details like names in response to our questions. We want to let you know that we intend that the results of the interviews will be published but we will do so in a way that does not identify you, so for example if you do use names, we'll remove those before any publications are made. So, please remember to keep

the discussions we have today private. Please do not share anything that is talked about today with anyone who is not present in the interview.

We really appreciate your time and willingness to speak with us. Do you have any questions before we get started?

Finally, before we start I'd just like to reconfirm that you consent to participate, and remind you that you can choose to withdraw your participation at any time. Are you happy to begin the interview?

**Family experiences with neurodevelopmental care. Ask all questions if possible.**
*Ensure answer captures parent/family perspectives on if CHD LIFE addresses patient barriers, is patient-centred/offers choice, easy to access, minimal burden, easy transitions between services/elements.*

I'd like to start by providing you with some context for our discussion today. The CHD LIFE Program has provided and supported developmental follow up for children with congenital heart disease after their surgery in different ways. For many families, the CHD LIFE care pathway started for your child when they were discharged from hospital after their open-heart surgery. Some children may also have had their development assessed or screened through the Program clinic before the pathway was developed.

1. Now, I'd like you to think back to when your child **first received care through the CHD LIFE follow-up** care pathway. Talk me through how that happened. *(Prompts: how long ago, how hear about/referred, was it hard to access?)*

2. Can you tell me about the developmental care and follow up your child has received through CHD LIFE?

3. What did you like about the support, care, or services you and your child received through CHD LIFE? *(Prompts: accessibility, availability, timeliness, appropriateness, type of care/support delivered, tailored to them, holistic, cost, close to home etc)*

4. What things didn't you like about being followed up and supported through CHD LIFE? *(Prompts: access, cost, barriers etc)*

5. What has been your experience with the health providers you have seen for developmental follow-up care for your child? *(Prompts: GP vs child health, tertiary vs local, skilled?)*

6. Thinking now about developmental follow-up since leaving the hospital after your child's early cardiac surgery, how would you prefer follow-up to be provided? *(Prompts: where and by who: come to Brisbane vs closer to home, by local GP, by the same person each time, telehealth etc)*

7. Part of the CHD LIFE care pathway is about **providing families with education** while they are in hospital. What was your experience with receiving education about the care pathway and/or developmental follow-up for your child? *(Prompts: did you receive it, was it useful, did you remember it, would you have preferred something different)*

8. Did you received education or counselling after you left hospital? If yes: from who and was it useful? If no: would you have like to and in what way?

<Reconfirm that the participant is happy to continue participating>

**CHD Life *patient/family Impact.* Ask all questions if possible – skip any already answered.**
Ensure answer captures parent/carer perspectives on CHD Life impact in terms of implementation, service, child outcomes.

9. Tell me about the impact that your child receiving care through the CHD LIFE pathway has had on you and your child? *(Prompts: what benefits have you seen: health, quality of life, family, education, referrals to other services, earlier follow up)*

10. How much do you think receiving care on the CHD LIFE pathway had an impact on your knowledge and understanding about neurodevelopmental follow-up for CHD? *(Prompt: not at all, a little, a lot)* Why?

11. How did you feel about the support/care/services you received through the CHD LIFE pathway? *(Prompt: satisfaction)*

12. What would you have done/what would have happened if your child hadn't received care via the CHD LIFE pathway? *(Prompt: would you have known about the importance of follow-up, would you have been able to access care, would you have travelled to Brisbane?)*

Now think about your **access to and use of health and community services**[3]. For example, having access to services and providers that meet your needs when you need them like your GP, child health, hospitals, specialists, and education services.

13. What types of services have you accessed as part of the care pathway?

14. Why did you access those? *(Prompts: referred by child health, for assessment, behavioural issues)*

15. How easy or challenging do you find it to access these services when you need them?

16. How has your access to and use of these services changed by being a part of the care pathway?

***CHD LIFE patient/family opportunities.*** **Ask all questions if possible – skip any already answered.**

17. Can you suggest any ways in which the follow-up of your child for neurodevelopmental concerns could be improved? *(Prompt: new models of care/services, access, scope)*

18. Do you have any questions for me?

Thank you for participating in the interview today. Please feel free to get in contact with the research team if you have any questions.

[end of interview]

Evaluation of CHD LIFE Neurodevelopmental Long-term Follow up Program (CHD LIFE)
**QUT Ethics Approval Number xxxx**

# Semi-Structured Interviews with Service Providers: Outline

**Aims:**

- Understand how the CHD LIFE cardiac developmental long-term care pathway was implemented, and how staff at all levels responded, both cognitively and behaviourally
- Highlight contextualised barriers and facilitators to the implementation and delivery of the CHD LIFE care pathway throughout Queensland
- Assess implementation outcomes such as adoption of care pathway activities by providers, and perceived fit of service with family and staff needs
- Assess relevant service outcomes such as increased timeliness or access to care, improved provider skills/knowledge and onward referrals to other services

**Structure:**
The interviews will be semi-structured, with open-ended questions which are adaptable to reflect the responses being provided. The conversation should explore issues as they are raised, with guidance and prompting as required. Interviewers/facilitators will ensure discussion progresses in a timely, yet informative manner. Interviews will last no more than 60 minutes via Zoom, Teams, or phone.

**Participants:**
Interviews will be held with key stakeholders involved in the management, funding, planning, implementation, or service delivery of the CHD LIFE care pathway.

**Process/Content:**
Review aims of interview and participant information sheet, particularly confidentiality, consent, privacy and right not to participate. Check for consent or e-consent. Begin recording and ask for verbal consent if not yet provided (virtual/phone). A guide for the content and design of questions is provided below. All questions in Section 1 should be asked but Sections 2 and 3 may need to be adapted based on the stakeholder role and their familiarity with CHD LIFE.

The content of the interviews is based on (a) capturing established implementation and health service outcomes and (b) examination of barriers and enablers underpinned the Consolidated Framework for Implementation Research[1,2]. CFIR is a widely cited and rigorously developed implementation determinants framework that was developed through a process of consolidating earlier published literature. It has a focus on how the inner setting, outer setting, intervention characteristics, individuals, and implementation processes influence implementation outcomes such as reach, appropriateness and effectiveness.

**References**

1. Damschroder LJ, Aron DC, Keith RE, Kirsh SR, Alexander JA, Lowery JC. Fostering implementation of health services research findings into practice: a consolidated framework for advancing implementation science. Implementation science. 2009 Dec;4(1):1-5.
2. Damschroder LJ, Reardon CM, Widerquist MA, Lowery JC. The Updated Consolidated Framework for Implementation Research: CFIR 2.0. Research Square (preprint). 2022. DOI: https://doi.org/10.21203/rs.3.rs-1581880/v1

# Semi-Structured Interviews with Project Stakeholders: Proposed questions

**Before beginning the interview**

| Location/Setting | Y/N | Notes |
|---|---|---|
| *Is the location private?* | | |
| *Is the location neutral for all participants?* | | |
| *Have accessibility considerations been addressed?* | | |
| *Have all WHS procedures been addressed?* | | |
| *Are there any other considerations to be noted for records prior to conducting the interview/focus group?* | | |

**Introduction**
Hi, my name is [________]. I'm speaking with you today because we are interested in your thoughts about neurodevelopmental follow-up care for children with congenital heart disease, and specifically the CHD LIFE Program. The information you provide to us will be used to help evaluate the CHD LIFE cardiac developmental long-term care pathway and to improve how it is delivered across the state.

There are no right or wrong answers and any information you can provide will be helpful. We won't criticise you or your opinions. Don't worry if you feel you cannot respond to some questions. It is fine to let me know you are unsure or do not know.

We will record the conversation with you if that is OK and will only share your conversation with other members of the research team or by making sure other people cannot identify your responses. We take your privacy and confidentiality seriously. We don't need you to use any specific details like names in response to our questions. We want to let you know that we intend that the results of the interviews will be published but we will do so in a way that does not identify you, so for example if you do use names, we'll remove those before any publications are made.

We really appreciate your time and willingness to speak with us. Do you have any questions before we get started?

**Section 1: Knowledge and experience with neurodevelopmental follow-up for CHD**

1. To start, I was hoping you could tell me a bit about your role here at [ organisation ] as it relates to care and neurodevelopmental follow-up of children with congenital heart disease?

2. How would you describe your knowledge about the risk of congenital heart disease on a child's neurodevelopment? *Probe: why do you say that? Where did you gain that knowledge?*

3. Talk me through how you might care for a child with congenital heart disease or who's had open heart surgery for congenital heart disease once they are referred to you/come into your care? [this could be a real or hypothetical case depending on who is being interviewed]. *Prompts: would you perform assessment/surveillance, provide education, know where to refer for other services?*

**Section 2: CHD LIFE implementation, barriers, and facilitators**

4. When and how did you learn about/become involved with the CHD LIFE care pathway?

5. Why did you engage with the CHD LIFE care pathway/why was it chosen for implementation in your setting/hospital/practice/area?

**[If they are not involved with CHD LIFE frame Question 6 as general neurodevelopmental follow-up care and then skip to Question 15]**

6. Think about your experience implementing the CHD LIFE care pathway/delivering services to families. What has worked well? ….What have been some of the challenges?

   *Prompt to ensure they talk about both what works well, and what the challenges are in current implementation/service delivery. Key domains to cover are*:
   - **Characteristics of the model of care** such as evidence-base, advantages over other options, adaptability to local context, complexity, supports/resources/materials, costs
   - **Outer setting of implementation** such as local sociocultural attitudes and values, local economic/environmental/political conditions, partnerships and connections, policies and laws, external pressures to implement (advocacy, competition)
   - **Inner setting of implementation** such as infrastructure (physical/IT/workflows), relationships and teams within organisation, communication within organisation, culture of organisation (e.g patient centred, equity, learning), tension for change, fit with existing systems, available resources (funding, space), access to knowledge/training
   - **Individuals** such as leaders, facilitators, implementation team members, implementation support. Also having capability, knowledge, motivation and opportunity to implement CHD LIFE.
   - **Processes such as** considering context, planning, tailoring, engagement, reflecting and evaluating.

7. Can you describe any ways you've had to adapt CHD LIFE care pathway activities or processes to fit your local context? *(Probes: what were the reasons for these adaptions? How easy was this adaptation?)*

**Section 3: Outcomes and impact of CHD LIFE**

8. In your opinion, what is the impact of the CHD LIFE care pathway on children and families? *(prompts: consider positive and negative impacts – early intervention, access to supports, well-being)*

9. How do you think the CHD LIFE care pathway addresses barriers to care for families? (*Prompts: access, knowledge, cost, travel)*

10. How much do you think that the CHD LIFE care pathway had an impact on your knowledge and understanding about neurodevelopmental follow-up for CHD? (prompt: not at all, a little, a lot) Why?

11. How do you think that the CHD LIFE care pathway has impacted your ability to support children with congenital heart disease and their families? Why?

12. How do you feel about being a part of the CHD LIFE care pathway? *(prompts: satisfaction/ did you like it?)*

13. What do you think are the key factors important for the care pathway's success or sustainability?

14. Can you suggest any ways in which the CHD LIFE care pathway could be improved?

15. What would be the best way to support you in the neurodevelopmental follow-up of children with CHD in the future?

16. Do you have any questions for me?

Thank you for participating in the interview today. Please feel free to get in contact with the research team if you have any questions.

[end of interview]

## Additional File 4

Supplemental Tables 2-7 present six categories of suggestions for improving the CHD LIFE follow-up care pathway. While all these suggestions are important to consider in terms of designing effective and appropriate decentralized models of follow-up care, it should be noted that number of suggestions made by parents or providers may not be feasible within current CHD LIFE program resourcing, geographical context and pathway structure. Any improvements should also align with the overall program philosophy to advocate for families to have improved care coordination and navigation within their own communities.

**Supplemental Table S2. Establish formal touchpoint/s for providing ongoing pathway support and navigation advice**

| Why is it needed? | What did parents and providers say it could it look like? | What could it improve? | Quotes |
|---|---|---|---|
| Families are struggling to know who to contact for neurodevelopmental follow-up support<br><br>Parents who did contact CHD LIFE team members reported the benefits of this<br><br>Not all children on the care pathway are eligible for existing care navigation/connected care approaches<br><br>Would be particularly useful for families outside of Brisbane or those with older children and access to fewer supports<br><br>Caregivers with certain characteristics and circumstances may need additional support | • A single person or team<br>• A care navigator or case worker – based either at the tertiary centre or locally to child<br>• Available online, via the phone or in person<br>• Act as touchpoint for both providers and patients seeking information about pathway implementation and services<br>• Help to navigate eligibility criteria, explain options for follow-up, or find and access the correct service<br>• Offer a directory of services, providers and resources<br>• Remind families when they are due for screening based on pathway key timepoints | • Continuity of care for families<br>• Have a trusted contact point for families to reach out to with concerns<br>• Assist with navigating the health system<br>• Adherence to care pathway<br>• Could reduce stress on families<br>• Families could have faster and easier access to information to navigate system and find relevant supports/services | *“It would have been handy to have someone to call, the middle ground kind of person to be like: our GP doesn't know if this is connected to his heart what do you think?” [F19, parent]*<br><br>*“To just hold your hand because it’s really bloody hard.” [F21, parent]*<br><br>*“We had a nurse navigator and they're good at helping you navigate the appointments, but they don't really do much else. Like you actually need someone, like a care person to walk alongside the family.” [F06, parent]*<br><br>*“Just at those critical points having people to reach out to.” [F15, parent]*<br><br>*“You haven't necessarily got anyone you can even call. And it's a sort of no man's land that there isn't any way of getting information.” [P106, GP]* |

**Supplemental Table S3. Provide check-ins or follow-up with tertiary staff after hospital discharge**

| Why is it needed? | What did parents and providers say it could it look like? | What could it improve? | Quotes |
|---|---|---|---|

| | | | |
|---|---|---|---|
| Parents want information but are often overwhelmed when they receive it in hospital and may forget it. Having information longer-term/later was perceived as more useful.<br><br>Parents feel too much onus is placed on them to drive follow-up and would like to share care more<br><br>Parents would like to feel supported in an ongoing way | Several options were proposed about how CHD LIFE could provide ongoing follow-up after discharge in a more structured manner:<br>• Check-in with parents some months after discharge to re-explain the pathway and provide further education<br>• Contact parents by letter/call/email/text annually to briefly check concerns, let them know any upcoming key screening time points based on child's age and how to access services for this, and/or provide education or support resources<br>• More formally embed discussion of development and the pathway into the process of hospital-based cardiology review e.g. have Cardiologists check-in with parents about how they are finding the pathway/any concerns | • Continuity of care for families<br>• Better timing of information<br>• Increase knowledge and awareness of parents about pathway and potential developmental issues<br>• Have a trusted contact point for families to reach out to with concerns<br>• Assist with navigating the health system<br>• Adherence to pathway<br>• Would reduce stress on families<br>• Families would have faster and easier access to information to navigate system and find relevant supports/services | *"By 2-3 years down the track it's all disappeared out of my head. I would have liked more support there as it's just so overwhelming at the time." [F02, parent]*<br><br>*"Even if it's just every year or two to say, 'Hey we're still thinking of you. Remember if you ever need anything there's these contact numbers and there's these resources available to you." [F17, parent]*<br><br>*"People live busy lives and even people that are on top of it might need some kind of help. The thing is he [child] actually fell through the cracks really hard with the one-year-old one, he didn't get that until he was 14 months old." [F01, parent]*<br><br>"*Make it usual practice for us as part of that clinical assessment in the [Cardiology] clinic to look at the neurodevelopmental follow up. … how their neurodevelopment is going. Are they on the pathway? Who's seeing them?" [P32, cardiologist]* |

**Supplemental Table S4. Awareness and capacity building of parents and providers**

| Why is it needed? | What did parents and providers say it could it look like? | What could it improve? | Quotes |
|---|---|---|---|

| | | | |
|---|---|---|---|
| Parents need to increase skills in health literacy and health navigation if they are to effectively drive care<br><br>Parents need information at the right time. It needs to be practical and tailored to their child. They often don't know what they need until they need it.<br><br>Providers require increased awareness of the care pathway and access to information about how to care for children with CHD and developmental concerns | Family education/support groups or family-based interventions to increase health literacy and related skills<br><br>A parent education day<br><br>Creation of new resources or sharing of existing resources in a more accessible way (Red Book, online, or as fact sheets) for families and providers:<br>• Information specific to development of children with CHD and things to look out for such as milestones and red flags<br>• Practical things parents can do with their child to help development<br>• More information about the care pathway itself | • Better education to increase knowledge and awareness of developmental concerns<br>• Better knowledge and awareness of the CHD LIFE care pathway and how it can support follow-up | *"It's just such an overwhelming time as a parent, I wish I had something solid." [F22, parent]*<br><br>*"A handbook for these kids with particular things to lookout for." [F17, parent]*<br><br>*"There's lots of resources around it's just how do you find them?" [F15, parent]*<br><br>*"Health services have an important role in providing that capacity, building linkages, information sharing, not just to local services but to families as well." [P47, hospital manager]*<br><br>*"We really need it [a resource] because one of the most common questions that families ask, where can I go to get all the stuff?" [P42, hospital manager]* |

**Supplemental Table S5. Provide greater structure and clarity about pathway for parents and providers**

| Why is it needed? | What did parents and providers say it could look like? | What could it improve? | Quotes |
|---|---|---|---|
| Not all parents knew what the CHD LIFE care pathway was, what the parts were, and if they were using it appropriately for their child<br><br>Parents felt the pathway needed more formalisation and structure, especially as children get older (>2 years)<br><br>Parents did not know who to contact for support<br><br>Pathway knowledge of providers and implementation in community was variable | • More information about the care pathway for parents<br>• A list of services and supports available<br>• Engagement and education with services and providers about their role in the care pathway<br>• Better clarification and education about role of GPs<br>• Support hospital and health services with more practical guideline implementation or standards to tailor and implement CHD LIFE care pathway within their region | • Better knowledge and awareness of the CHD LIFE care pathway and how it can support follow-up<br>• Better implementation of care pathway across providers, services and regions<br>• Adherence to care pathway | *"Explaining exactly what this is and what you can access, who to phone if you have a question, that sort of thing. I don't know if that was available to me somewhere and I just missed it?" [F12, parent]*<br><br>*"We're given a lot of information at the time so it's hard to remember which was exactly related to the pathway." [F04, parent]*<br><br>*"I didn't receive something to say you know this is what it is and this is when you should be getting the checks and these are the checks that should be done. There was a lot of Googling trying to work out what was meant to be done and when." [F22, parent]*<br><br>*"Now he's bigger [child health] is not really for us so I don't know where I'd go for his next one to be honest." [F01, parent]*<br><br>*"Probably more clear specific guidelines around what the program needs each HHS to implement…like be a bit more clear." [P66, nurse]* |

**Supplemental Table S6. Improved communication, engagement and coordination between specialists and local services/hospitals**

| Why is it needed? | What did parents and providers say it could look like? | What could it improve? | Quotes |
|---|---|---|---|
| Variable implementation of pathway across services and regions<br><br>Perception that community-based providers have less specialist knowledge<br><br>Challenges with health service navigation<br><br>Electronic health record (including care pathway alert) not available statewide or to private services | • Increase engagement with hospitals and health services across the state<br>• Increase engagement with non-health services e.g. schools<br>• Better education and communication with providers across the state<br>• Greater involvement of GPs and Pediatricians in the pathway<br>• Better use of nationally funded early intervention supports such as playgroups and childcare<br>• Greater visibility of CHD LIFE care pathway flag on medical records across primary care | • Providers more engaged in pathway<br>• Less need to be parent driven<br>• Better continuity of care<br>• Easy to navigate and access services<br>• Broader scope of services available | *"I think just having a really clear letter that says this person is part of that follow-up we need to keep a track of, at these landmarks is where the milestones will be checked…And if there's anything that's an issue, this is who you need to flag with it. That document gets scanned in the notes, little comment at the top of Best Practice or Medical Director." [P106, GP]*<br><br>*"But if we have these at-risk children flagged from the moment they leave hospital… cardiac babies that need to go on the cardiac pathway then yeah, that's a good starting point to make sure they're followed up properly." [P58, nurse]* |

**Supplemental Table S7. Mental health and well-being support for parents**

| Why is it needed? | What did parents and providers say it could it look like? | What could it improve? | Quotes |
|---|---|---|---|
| Parents spoke of being stressed about their child, appointments, work, childcare options, and of having trauma related to their child's diagnosis and surgery<br><br>Parents also mentioned feeling isolated, scared, overwhelmed and uncertain | • Facilitate connections between families in person or online<br>• Offer more formal peer support groups or opportunities to come together<br>• Provide mental health support, check-ins and referrals | • Better mental health support for parents<br>• Peer connections | *"I didn't really consider the emotional impact… It was just a very traumatic experience." [F05, parent]*<br><br>*"The health of the child is paramount but also the parents who have been through an extremely traumatic…I'm sorry, I still get upset." [F06, parent]* |

**Other improvements**

Other suggestions included:

- better understanding and implementation of processes for following up school-aged children and those who had aged out of some pathway services, including articulating the role of GPs and education providers in the pathway: *"The model we have is working very well up to school-aged children, we do need to make it sustainable throughout childhood adolescence." [P32, cardiologist]*
- considering whether the pathway could be broadened to include other groups who require regular follow-up or for all children with CHD if it improved sustainability and/or funding options: *"So, for regional centres, they may not see many CHD kids, but all of these kids we know have additional risk factors. How are we bundling these kids to make it more sustainable by having a larger population?" [P42, hospital-based manager]*
